# An Effective Gender-Affirming Care and Hormone Prescribing Standardized Patient Case for Residents

**DOI:** 10.15766/mep_2374-8265.11258

**Published:** 2022-06-03

**Authors:** Ben J. Hersh, Rebecca E. Rdesinski, Christina Milano, Rebecca E. Cantone

**Affiliations:** 1 Clinical Assistant Professor, Florida International University Herbert Wertheim College of Medicine and Care Resource Community Health Centers; 2 Senior Research Associate, Department of Family Medicine, Oregon Health & Science University School of Medicine; 3 Associate Professor of Family Medicine, Oregon Health & Science University School of Medicine

**Keywords:** Transgender, Family Medicine, Gender Identity, LGBTQ+, Standardized Patient, Diversity, Equity, Inclusion

## Abstract

**Introduction:**

It is estimated that at least 700,000 individuals in the United States identify as transgender or gender expansive. Many have confronted marginalization within the health care system, leading to suboptimal care and inequitable health outcomes. Health sciences trainees do not receive adequate training in gender-affirming care. The authors therefore created, piloted, and evaluated a formative standardized patient case for gender-affirming care for family medicine resident learners that could be given with limited resources in primary care and health professional education.

**Methods:**

The curriculum for the case was developed with patient input and with family medicine physicians skilled in education, simulation, and gender-affirming care. The first case was held for 20 residents in a 4-year family medicine program in the Pacific Northwest. Nineteen participants completed pre/post case surveys delineating knowledge, awareness, attitudes, and intended behavior regarding providing gender-affirming care.

**Results:**

Self-reported knowledge and awareness increased after standardized patient case participation in multiple skill areas related to providing gender-affirming care. Faculty observers informally reported that the session increased their knowledge and comfort as well.

**Discussion:**

Implementation of this gender-affirming standardized patient case inclusive of community input was associated with successful improvements in self-reported measurements of resident knowledge and awareness of providing gender-affirming care. Additional institutions should consider such training to improve health care equity for this population.

## Educational Objectives

By the end of this activity, learners will be able to:

1. Use a patient's affirmed name and pronoun.
2. Demonstrate a history and exam relevant to gender-affirming care.
3. Formulate a hormone prescribing and monitoring plan with their preceptor.
4. Execute an informed consent for hormones and shared decision-making on a prescribing plan with their patient.

## Introduction

At least 700,000 individuals in the United States identify as transgender.^1^ Many have faced significant marginalization in settings including health care.^2^ Respondents to the 2015 U.S. Transgender Survey reported increased exposure to unemployment, violence, social isolation, suicidal thoughts, substance use, and HIV compared to the general population.^2–5^ In health care environments, transgender individuals encountered refusal of services, harassment, poor access, and lack of practitioner knowledge, with one in four not seeking care. Further, 31% reported their health care practitioners were unaware of their gender identity. Almost one-quarter had to teach their practitioner about transgender people to receive appropriate care, and only half received hormone-based therapy, though almost 80% desired it.^2^

Training deficits to provide care for this population are well known, and efforts have continued to integrate LGBT health into health care education.^6^ While only 16% of institutions have significant transgender curricula, they note positive changes in learners’ knowledge, attitudes, and beliefs.^7,8^ Historically, half of surveyed institutions have had no LGBT training,^8^ and in 2021, although 72% of family medicine clerkship directors agreed it should be required, only 26% reported being comfortable teaching it.^9^ Even comprehensive gender clinics, in 2022, are asking for more primary care providers to care for transgender patients.^10^ Within *MedEdPORTAL,* current transgender curricula include cancer screening,^11^ preclinical training,^12,13^ sexual history-taking,^14^ communication,^15^ LGBTQ-wide discussions,^16,17^ and multiple-session medical school curricula.^12,13^ One resource tackles a standardized patient (SP) scenario,^18^ but none specifically include practicing hormone prescribing within an SP case. We believe that experiential learning in a safe environment is a unique formative experience in which to practice an informed consent hormone-initiation model addressing the patient-reported gaps in provider knowledge.

Family medicine residencies are essential for teaching skills in gender-affirming care to address such education gaps and health care inequities. Residents in this program have an introductory lecture about gender-affirming care, but few are able to practice this care clinically with real patients early in their training. We aimed to develop and evaluate a formative SP case on gender-affirming hormone initiation for residents at a 4-year academic family medicine program in the Pacific Northwest, with the goal of improving residents’ knowledge, awareness, attitudes, and intent regarding providing gender-affirming care.

## Methods

### Case Development

After institutional review board approval (#00017781, Oregon Health & Science University, December 5, 2017), we crafted the learning objectives and case scenario through collaboration with members of the local transgender community and iterative review by educators experienced in transgender health, simulation, and education. We garnered community input through an anonymous survey and a focus group to identify priorities for an “establish care visit.” These priorities became the learning objectives: using a patient's affirmed name and pronouns, demonstrating a history and exam relevant to gender-affirming care, formulating a hormone prescribing and monitoring plan for a patient desiring hormones, and executing a hormone informed consent and shared decision-making on a prescribing plan with the patient (Appendix A). During this time, we also attempted to recruit SPs identifying as members of the LGBTQIA+ population.

Prior to the day of the case, we gave the SPs the case scenario (Appendix B) and invited them to a 1-hour presession training with an SP educator and the case authors. The training included a full read of the script, addressed questions and concerns about the materials, discussed the focus for feedback on communication skills, and allowed SPs to choose which hormones they planned to ask for during the scenario. Faculty serving as observers for checklist items did not have to be experienced in gender-affirming care but were required to attend a 20-minute training immediately before the case scenario began to orient to their role of observing residents in the room while completing a checklist. That training also included the residents and faculty who served as content experts, giving them a chance to discuss the workflow of the half-day. We held the SP case in the simulation center of an academic health institution and utilized trained SPs. We set up the rooms to be typical of an outpatient office visit, and no supplies were needed. Since content experts and faculty observers worked in tandem, no additional staff were required.

### Case Flow

All residents in our program were invited to this formative SP case. We gave resident participants a case information sheet and guided note template (Appendix C) and gave observing faculty a checklist (Appendix D) for the patient encounter at the beginning of the session. SPs gave learners an exam card (Appendix E) when prompted. Content experts had computers available to access the online resources of their choice, as well as paper copies of reference materials. These included resources on gender terminology and definitions, primary care, and hormone evaluation as noted in the online learning module Improving Care for Transgender People,^19^ a summary of disparities found in the 2015 U.S. Transgender Survey,^2^ and informed consent forms found through Fenway Health.^20,21^ We did not attempt to cover all details of hormone follow-up plans given that this was a short intervention and that such materials were covered elsewhere in the residents’ curriculum.

The first case delivery included 20 residents (nearly half of all residents, all years of training), 10 faculty observers (not content experts), six content experts (faculty or residents), and 10 trained SPs. The afternoon included a brief introduction to the session's schedule; 15 minutes to collect a history from an SP and ask for an exam; 15 minutes to review guidelines and discuss with a content expert; and 15 minutes to obtain additional information, complete an informed consent, and discuss a plan. Participants received 5 minutes of individual feedback from the SP and then debriefed as a large group with a discussion of teaching points. The debrief included residents sharing what they had learned and a review of the materials made available during the guidelines review. Prompts included what resources the residents found most helpful, what they learned about communication and terminology, and what changes they would make in their clinical practice having reviewed the guidelines and performed an informed consent. We staggered two groups of 10 residents each in and out of the simulation rooms to maximize the number of participants in a shorter time with fewer SPs and faculty.

### Learner Assessment

We created a checklist catered towards the priorities our transgender community had identified as important knowledge areas for a first visit. These included obtaining the appropriate history points, asking only for relevant exams, asking about plans for gender-affirming care, discussing initial prescriptions of hormones, and completing the informed consent. SPs gave behavioral feedback on communication and listening skills, describing how they felt as a patient, without a checklist. Faculty observers reviewed the checklist items and gave the checklist to the learner for review after they had completed the scenario.

To study the effect of this intervention, we gave participants a paper presurvey before the case scenario and a postsurvey at the time of the debrief (Appendix F). The surveys measured four domains—knowledge, awareness, attitudes, and intended behavior—and utilized 5-point Likert scales measuring levels of confidence, agreement, and likelihood. We analyzed responses to gauge if there were differences before and after the case regarding these domains using the nonparametric Wilcoxon signed rank sum (WSRS) test. We utilized medians and distributions for analysis, and means were also reported to capture changes (Table). Since multiple hypotheses were tested, we calculated Benjamini-Hochberg (BH) critical values using a false discovery rate of .05 and compared each survey item's *p* value with its corresponding BH critical value to determine significance more accurately than could the WSRS alone.

**Table.**
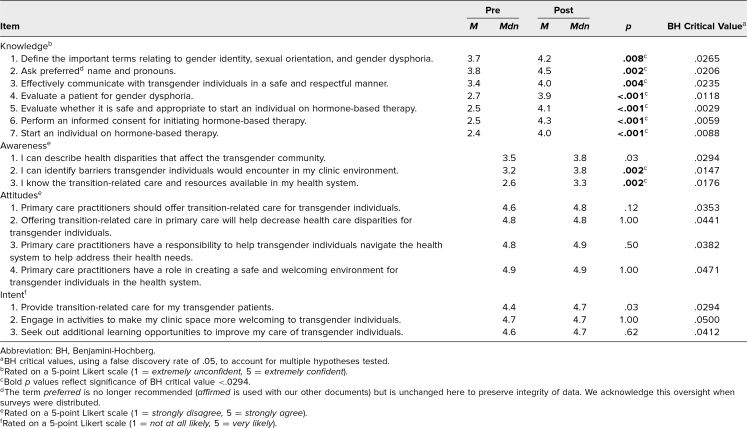
Comparison of Survey Items: Pre to Post

## Results

Nineteen of 20 participants representing all four classes of residents in one academic family medicine program completed both surveys. Data represented Kirkpatrick level 1.^22^ Items showing attitudes and intent began with the highest values possible and remained unchanged (Table). Self-reported knowledge and awareness showed increased medians and means for asking/using affirmed name and pronoun, evaluating a patient for gender dysphoria (demonstrating the history and exam), discussing the safety of hormones, executing an informed consent, and formulating a plan for hormones. Residents reported increased awareness of barriers to care and transition-related resources and demonstrated statistically significant improvement on multiple measures. Mean values are also reported to show any pre-to-post changes.

Faculty observers similarly noted that they felt they had learned from the scenario as well. In regard to performance, we did not collect learner checklist data as the checklists were returned to the residents to review for their own education.

## Discussion

The curricular goals of this SP case were paired to match the needs of our community, and we observed successful improvement of the identified top priority of using affirmed names and pronouns. There were significant improvements in self-reported knowledge and attitudes as well, which we hope will lead to residents reducing health care disparities in their future practices. The high presurvey scores in attitudes and intended behavior confirmed the sense of responsibility to serve the transgender community, yet growth still occurred. Family physicians should aspire to provide competent care for all patients, regardless of identity, and this training can be replicated at any residency program.

During curriculum and SP development, we encountered the challenge of providing an authentic experience for learners. We grappled with the moral dilemma of having cisgender actors portray a transgender identity and having transgender/nonbinary actors seek hormones that might not match their identity. While we recruited SPs identifying as LGBTQIA+, education was needed to keep the case standardized, in lieu of sharing personal experiences, given care the SPs may have received as patients. This was also addressed by allowing all SPs to pick the hormones (estrogen or testosterone based) that felt safe for them and their experiences. While our simulation center was supportive of this education, challenges in acceptance and hiring of transgender individuals may create barriers at other institutions.

Since this curriculum was written, we have recognized the role that we in health care have in perpetuating gender-binary stereotypes in relation to phenotypic features with the use of words *masculinizing* and *feminizing.* We now recommend using gender-neutral language for hormone therapy, specifically, naming the hormones involved (i.e., testosterone therapy and estrogen-centered therapy) without attributing the effects of those hormones to any specific gender. Future users at other institutions should be certain to review terminology to reflect the ongoing changes specific to the LGBTQIA+ community. Throughout case development, we needed to alter the language we used to reflect the changing climate and, as in the Table, learned the importance of consistency between all materials disseminated to learners to send a clear message on language to use.

Limitations include that this was a brief curricular intervention with a small number of residents. The absence of checklist data was initially intentional to focus on learner development, but utilization of the checklist should be considered to obtain more information on learner performance. Additionally, while these family medicine residents already had interest in this education, this may or may not be true of other residents. In the future, we hope others can reproduce this experience to gather multiple cohorts of data when curricular space and simulation resources are available. However, the structured nature of the scenario with the available references provided could offer a safe opportunity to learn with minimal faculty expertise in any environment and across other institutions or programs that may be interested.

Given the continued need for gender-affirming health care training, this SP case is a simple intervention to increase skills of any learner with minimal expertise. Smaller institutions can sequentially run a scenario without faculty observers and with just one faculty member accessing resources. A train-the-trainer option could lead to more educators feeling confident, which could in turn expand training and eventually improve patient care. Future work on and expansion of the number and diversity of learners’ experiences are encouraged. We hope this low-resource SP case scenario with high educational value can be of assistance to other learners and institutions.

## Appendices


Standardized Patient Case Development Tool.docxStandardized Patient Case Scenario.docxParticipant Case Materials.docxObserver Checklist.docxPhysical Exam Results.docxPre-Post Survey.docx
*All appendices are peer reviewed as integral parts of the Original Publication.*
