# Supplementary material for: An Effective Gender-Affirming Care and Hormone Prescribing Standardized Patient Case for Residents
Source: MedEdPORTAL. 2022 Jun 3;18:11258. doi: 10.15766/mep_2374-8265.11258 (PMC9163229; doi:10.15766/mep_2374-8265.11258)
Supplement: Supplementary file 1 — Standardized Patient Case Development Tool.docxStandardized Patient Case Scenario.docxParticipant Case Materials.docxObserver Checklist.docxPhysical Exam Results.docxPre-Post Survey.docx [file mep_2374-8265.11258-s001.zip › B. Standardized Patient Case Scenario.docx]

**Case Title:** Establish Care Visit

**Case Author:** Ben Hersh, Rebecca Cantone, Christina Milano

# Time Allotted for Case: 1 hour

**Framework**: Patient coming in to establish care with a new primary care provider, interested in hormone therapy for gender affirmation.

**Objectives**: Learners completing this standardized patient case will demonstrate the ability to:

- Respectfully interact with patients, including affirmed name and pronoun use
- Collect an appropriate history including the key components needed to consider hormone initiation
- Perform an appropriate physical exam
- Utilize provided Transgender Health Primary Care recommendations to create an appropriate assessment and plan
- Complete an Informed Consent
- Communicate next steps to the patient

**Competencies**: Learners will demonstrate skill level in the following competencies by OHSU SOM/ ACGME FM Residency (paraphrased) including:

- PCP 1/PC-1: Gather essential and accurate information about patients and their conditions through history taking, physical examination, review of prior data and health records, laboratory data, imaging and other tests.
- PCP 5/PC-3: Partners/personalizes healthcare services, prevention and promotion of health
- ICS 1/SBP-3: Communicates with patients/public with diverse socioeconomic/cultural backgrounds
- PPPD1/PROF-3: Humanism/cultural proficiency with diverse patients

**Room set up**: One patient chair/exam table and a wheeled stool for the learner. **Props for the room**: Stethoscope provided, guidelines provided (paper or computer) **Dress for SP**: Work scrubs (to be provided).

| Case setting | Family Medicine outpatient office |
| --- | --- |
| Patient name | Alex Jimenez |
| Age of actor | Able to play someone 21-49 years old |
| Gender | Identifies as gender non-binary. Individual SPs will select whether their character would like to undergo masculinization or feminization in consultation with the proctors. |
| Ethnicity | Can reflect the background of the individual SP |
| Occupation | Dental Hygienist |

**CHARACTER**: Alex Jimenez, 21-49 years old (pick an age that you best portray)

**SETTING**: A primary care clinic. You have recently obtained health insurance and are here to establish care with a new clinician. Your main goal for the visit today is to initiate masculinizing or feminizing hormone therapy as a gender diverse individual.

**Affect**: Nervous and worried, trying to appear calm (but failing).

You have never spoken with a medical person directly about this concern before, but have experienced embarrassment and invalidation during interactions with the medical system in the past. You identify as non-binary and have other friends who identify as Trans who have also felt invalidated in medical offices, had inappropriate physical exams performed, or told that they shouldn’t be Trans or that “it’s a choice.”

# Background for Actor: You have been living openly as a non-binary person for several years. You have been contemplating medical transition for at least a few years, are very familiar with the anticipated effects and risks (you have both researched the subject and have friends who are on hormones) and feel fully ready to initiate hormone therapy.

**Lack of medical coverage has been holding you back.**

You want to start hormones as soon as possible, though understand this may not be a possibility today, and that the clinician will likely need to do some laboratory work first.

You do not have any other concerns or needs from the provider today [in case the provider does good agenda-setting].

You answer questions easily and freely if you feel they have made you comfortable to do so, but avoid eye contact and give one word answers if they are making judgements or unclear comments/questions.

**Dress**: Work scrubs (to be provided). Aim for gender neutral appearance. Suggestions include wearing hair natural or slightly messy, minimal make up, dressing in neutral tones (black, white, grey), and wearing a larger, less fitted top.

**OPENING LINE**: I’m here today because I finally got health insurance with my new job and want to start working with a provider on getting hormones. I heard from a friend this is a safe place to talk about it and where they have been prescribed hormones.

**CHIEF COMPLAINT**: Establish care. Start hormone therapy. Patient/Actor detailed information:

Affirmed Name: Alex

Pronouns and Gender: As above, you identify as gender non-binary and use they/them pronouns.

You have felt something was unaligned with your designated sex since you were in elementary school, but only became familiar with the concept of gender transition about 10 years ago. You have been working with a therapist for the past few months and are confident in your gender identity and your desire to start medical transition. You have discussed your plans with your partner Sam (also gender non-binary if they ask), your family, and your friends who are all very supportive.

HORMONES: You are not currently taking gender-affirming hormones and have not taken them in the past. You are interested in starting hormones as soon as possible. You have close friends who have transitioned with hormones and don’t have any specific questions.

You would like to start hormone therapy at the lowest recommended dose.

If you are seeking masculinization: You would like to start “the testosterone injection.”

If you are seeking feminization: You would like to start “the estrogen that dissolves under the tongue.”

SURGERIES: You have not had any gender affirming surgeries/treatments in the past. You may be interested in surgeries one day, but not now. (see educational supplement for surgery descriptions)

**REVIEW OF SYSTEMS:** All negative, other than a history of anxiety, which is well controlled without medication.

# PAST MEDICAL HISTORY

GENERAL HEALTH

You do not have any known medical conditions.

ALLERGIES/MEDICATIONS

No allergies. You take a daily multivitamin.

MENTAL HEALTH

You have a history of anxiety, which is treated with physical activity and therapy (low cost through a local advocacy organization). You have not required medication. You do NOT have any history of:

Depression Suicide attempt/Self harm

SOCIAL HEALTH

You currently live with your partner Sam, who also identifies as gender non-binary, and has had a hysterectomy (designated female at birth). You are in a monogamous relationship. You are currently co-parenting 2 children with Sam. Your current living situation is safe and you have never experienced any form of physical, verbal, or sexual abuse in the past. You have a new job as a dental hygienist and have recently come out as non-binary at work, which was well received.

You do not use tobacco/nicotine products, rarely drink alcohol (“maybe 2 drinks a month”) or use illegal drugs now or in the past.

FAMILY HISTORY

Your immediate family (mother/father/brother/sister) are healthy and do NOT have any of the following conditions:

Heart Disease or Stroke Cancers

Blood Clot Problems (DVT, PE)

High Blood Pressure High Cholesterol Diabetes

REPRODUCTIVE HISTORY

# For Designated Female at Birth:

Obstetric History: You have never been pregnant and will not be seeking future pregnancies or having additional children. You plan to remain monogamous.

Gyn History: First period was at age 11. You are still having regular monthly menstrual cycles, which cause you distress. You have remained up to date with pap smears which have been normal. Most recent was 2 years ago. You have never had a mammogram.

# For Designated Male at Birth:

Your partner Sam does not have a uterus nor do they produce sperm. You plan to remain monogamous. You do not plan to have additional children.

RESPONSE TABLE

| **Prompt** | **Your Response** |
| --- | --- |
| Introduced self to the patient | Prompt them to introduce themselves if they haven’t. |
| Used words/terms understandable to the patient | If they use complex words without explaining what they mean, do not ask for definitions. Make sure to mention which words caused confusion during feedback. |
| Showed listening body language (leaning forward, looking at patient) | If the participant is too close for your own comfort scoot back or lean back. |
| Used empathetic techniques (repeat feelings, legitimize concerns) | No response required. |
| Asked about patient's concerns and questions | State you have friends who are on hormone therapy, so you have a good sense of the risks, time tables, follow up. |
| Assessed patient's understanding (ex: asked patient to repeat statements, asked what patient knew, etc.) | If you are asked to repeat what you heard, try to summarize what they told you as accurately as you can. |
| Clearly communicated to patient what is to be done after this appointment | You will be fine with any plan they suggest. |
| Appropriately admitted uncertainty, and, if applicable, offered to get more information for patient | No verbal response required – you feel it is a sign of a good doctor if they need to look up the most current recommendations. |
| Asked about patient's preferred terminology for their anatomy | You state you are comfortable using the standard medical terms. |
| Asked to have an exam performed | Ask what parts of the exam the participant would like to perform. Decline, if participant declares intent to perform breast, genital, or pelvic exams. Give exam form. |
