# Supplementary material for: An Effective Gender-Affirming Care and Hormone Prescribing Standardized Patient Case for Residents
Source: MedEdPORTAL. 2022 Jun 3;18:11258. doi: 10.15766/mep_2374-8265.11258 (PMC9163229; doi:10.15766/mep_2374-8265.11258)
Supplement: Supplementary file 1 — Standardized Patient Case Development Tool.docxStandardized Patient Case Scenario.docxParticipant Case Materials.docxObserver Checklist.docxPhysical Exam Results.docxPre-Post Survey.docx [file mep_2374-8265.11258-s001.zip › C. Participant Case Materials.docx]

Participant Scenario

Alex Jimenez is here to establish care with you and is seeking gender affirming care. You are meeting Alex for the first time today.

Your task at this station is to collect a thorough enough history to evaluate whether Alex is a candidate for hormone therapy.

When you are ready to perform an exam, ask permission to do so and state which parts of an exam you would like to perform and you will receive exam findings.

You will have 15 minutes to complete the first part of your visit.

After meeting with Alex, you will move to another room and have an additional 15 minutes to review guidelines (provided) to help you:

1. Determine whether Alex is a candidate for hormone therapy
2. Determine whether Alex has capacity to consent to hormone therapy (as you would for any other patient requesting medical treatment)
3. Select any additional laboratory tests you would like to order
4. Propose an initial hormone regimen
5. Schedule an appropriate interval for your next follow up visit

Once you have a developed a plan, you will have an additional 15 minutes to re-enter the room to discuss your plan with Alex and to review a consent form that states the risks and expectations of hormone therapy initiation. As always, use clear communication techniques.

—————

Vital Signs collected by support staff today:

Blood Pressure 111/76, Heart Rate 78, Respiratory Rate 15, Temperature 97.6F, BMI 24

—————

You reviewed Alex’s care exchange record prior to entering the room. Other than some urgent care encounters for upper respiratory infections, you do not see any known chronic conditions, surgical history, or active medications.

Labs collected during urgent care visits and a voluntary workplace health initiative about six months prior:

CBC WNL CMP WNL

A1C: 5.4

TSH: 1.56

Total Cholesterol: 164

Triglycerides: 85

HDL: 76

LDL: 88

HIV negative

Hep B sAB: positive Hep C AB: negative

LEARNER NAME:

PERTINENT MEDICAL HISTORY (Any known

history of the following):

Heart disease

Liver disease

Hyperlipidemia

Hypertension

Hx of DVT or PE

Diabetes

Cancer/History of Cancer

Tobacco/Nicotine use

Migraines

PRESCRIPTIONS (If any):

Plans for fertility

PERTINENT MENTAL HEALTH HISTORY

(Any known history of the following):

Depression

PTSD

Suicide attempt/Self harm

Bipolar disorder/Schizophrenia

Mental health hospitalizations

Substance Use

FOLLOW UP APPOINTMENT (When would

you like this scheduled):

PHYSICAL EXAM FINDINGS: As noted on

patient form.

DIAGNOSIS: Gender Dysphoria in Adult

CANDIDATE FOR HORMONE THERAPY? (Y/ N)

CAPACITY TO CONSENT? (Y/N) LABORATORY EVALUATION:

TODAY (Which would you like to order, if any):

FUTURE (Which labs would you like to order and when would you like these collected, if any):
