# Supplementary material for: An Effective Gender-Affirming Care and Hormone Prescribing Standardized Patient Case for Residents
Source: MedEdPORTAL. 2022 Jun 3;18:11258. doi: 10.15766/mep_2374-8265.11258 (PMC9163229; doi:10.15766/mep_2374-8265.11258)
Supplement: Supplementary file 1 — Standardized Patient Case Development Tool.docxStandardized Patient Case Scenario.docxParticipant Case Materials.docxObserver Checklist.docxPhysical Exam Results.docxPre-Post Survey.docx [file mep_2374-8265.11258-s001.zip › D. Observer Checklist.docx]

**Observer Checklist**

## Learner: Date:

*Place a check in front of each task that the learner accomplished correctly. Do not place a check for any tasks*

*Medical decision making:*

### STOP. If the case involved discussion of hormone therapy for feminization, continue below. Otherwise skip to checklist item 30.

**FEMINIZATION**

*Pertinent History: The learner assessed for history of:*

*that were forgotten, done partially or incorrectly. If "and" is*

*used for a task, the learner must complete all tasks. If "or" is used for a task, the learner must complete or address at least one of the tasks. Learner will have 2 minutes to “wrap-up” the*

*interaction, discuss follow-up, and/or provide closure without*

1. VTE or PE
2. Migraine
3. Tobacco Use
4. Cancer

*prompting.* *Please note this is an introduction to informed consent, and specific details of follow up and additional medications should be covered elsewhere.*

*Communication Skills: The learner:*

1. Introduced self to the patient

2. Elicited affirmed name and pronouns from patient (avoids term “preferred”)*****

3. Asked open ended questions about gender identity

4. Avoided undefined jargon

5. Showed listening body language (leaning forward, looking at patient)

6. Used empathetic techniques (repeat feelings, legitimize concerns)

7. Assessed understanding of the risks and expectations for starting hormone therapy

8. Asked about patient's concerns and questions using an open-ended question (e.g., “what questions do you have?” rather than “Do you have any questions?”)

9. Assessed patient's understanding (ex: used “teach back”, asked patient to repeat statements, asked what patient knew,

etc.)

10. Clearly communicated to patient what is to be done after this appointment

11. Appropriately admitted uncertainty, and, if applicable, offered to get more information for patient

12. Explained medical documentation requirements in patient chart (e.g., “gender dysphoria” diagnosis

*Medical knowledge: The learner:*

13. Determined that the patient would be an appropriate candidate for hormone therapy

14. Reviewed appropriate consent form

15. Discussed risks of hormone therapy

16. Discussed time of expected onset of effects of hormone therapy

21. Plans for fertility

22. Mental Health history

*Physical Exam: The learner:*

23. Performed an exam

*Laboratory: The learner ordered: Today:*

24. BMP (alternatively BUN, Cr, K+)

*3 months after hormone start:*

25. BMP (alternatively BUN, Cr, K+)

26. Serum Estradiol

1. Total Testosterone

*Hormone Therapy Plan: The learner prescribed********:*

1. Spironolactone 25-50 mg, QD - BID
2. Estradiol oral/sublingual 1-4 mg/day OR Estradiol transdermal 50-100 mcg

OR Estradiol ≤20 mg or Cypionate ≤2 mg IM q2weeks

*Follow Up Plan: The learner scheduled follow up:*

1. 1-3 months after hormone start

### STOP. If the case involved discussion of hormone therapy for masculinization, continue directly below with item 30. Otherwise skip ahead to “Comments for the participant.”

**MASCULINIZATION**

*Pertinent History: Assessed for history of:*

|  | 31. | Cardiovascular Disease |
| --- | --- | --- |
|  | 32. | Diabetes |
|  | 33. | Hyperlipidemia |
|  | 34. | Cancer |
|  | 35. | Plans for fertility |
|  | 36. | Mental Health history |

*Physical Exam*

37. Performed an exam

*Laboratory Today:*

38. CBC (alternatively HGB & HCT)

*3 months after hormone start*********:*

39. CBC (alternatively HGB & HCT)

40. Total Testosterone

*Hormone Therapy Plan*

41. Testosterone Cypionate or Enthanate 20-50 mg/week subQ (other doses may be acceptable)

*Follow Up Plan*

42. Check in 1-3 months after hormone start

* We recommend against the term “preferred” as learners may see this in electronic health records

# Comments for the participant:

**OVERALL**

1 2 3 4 5

Recommend significant education prior to independently caring for this population

Has some knowledge. Would benefit from additional resources to work with this population

No reservations. Recommend for independent practice with this population

## Evaluator Name:

## ******In conjunction with local experts, the authors synthesize multiple sources into our prescribing practices, placing a high priority on patient preference. We present all effects of each medication (including some not listed), and then defer to patient preference as to how to initiate, in combination, staggered, or as monotherapy. Users may change specifics of plans based on local and/or institutional practices and/or as evidence develops.

## *******In recent literature, estradiol is suppressed within 2 months with anovulation, so we do not recommend estradiol checks, though patient preference and others’ institutional recommendations may include this. Citation: Taub RL, Ellis SA, Neal-Perry G, Magaret AS, Prager SW, Micks EA. The effect of testosterone on ovulatory function in transmasculine individuals. Am J Obstet Gynecol. 2020;223(2):229.e1-229.e8.doi:10.1016/j.ajog.2020.01.059
