# Supplementary material for: An Effective Gender-Affirming Care and Hormone Prescribing Standardized Patient Case for Residents
Source: MedEdPORTAL. 2022 Jun 3;18:11258. doi: 10.15766/mep_2374-8265.11258 (PMC9163229; doi:10.15766/mep_2374-8265.11258)
Supplement: Supplementary file 1 — Standardized Patient Case Development Tool.docxStandardized Patient Case Scenario.docxParticipant Case Materials.docxObserver Checklist.docxPhysical Exam Results.docxPre-Post Survey.docx [file mep_2374-8265.11258-s001.zip › E. Physical Exam Results.docx]

PHYSICAL EXAM:

GEN: No acute distress, pleasant, somewhat anxious appearing

HEENT: Normocephalic, atraumatic, pupils equal

CV: Regular rate and rhythm, no murmurs, rubs or gallops

CHEST: Lungs clear to auscultation bilaterally ABD: + Bowel sounds, non-tender, nondistended

EXT: Warm, well perfused, without edema NEURO: No focal asymmetry, tremor, or movement disorders
