# Supplementary material for: An Effective Gender-Affirming Care and Hormone Prescribing Standardized Patient Case for Residents
Source: MedEdPORTAL. 2022 Jun 3;18:11258. doi: 10.15766/mep_2374-8265.11258 (PMC9163229; doi:10.15766/mep_2374-8265.11258)
Supplement: Supplementary file 1 — Standardized Patient Case Development Tool.docxStandardized Patient Case Scenario.docxParticipant Case Materials.docxObserver Checklist.docxPhysical Exam Results.docxPre-Post Survey.docx [file mep_2374-8265.11258-s001.zip › A. Standardized Patient Case Development Tool.docx]

Appendix A: Standardized Patient Case Development Tool

Date: March 20^th^, 2019

Primary Case Author: Ben Hersh, MD, Rebecca Cantone, MD

Secondary Case Author: Christina Milano, MD

Standardized Patient Educator: Rebecca Cantone MD and Ben Hersh MD

Name of Case: Establish Care Visit

Name of educational and or assessment activity: Gender-affirming standardized patient case

Patient Name: Alex Jimenez

Chief Complaint: Establish Care. Start Hormone Therapy.

Most likely Diagnosis and Differential with rationale from history and/or physical exam:

Gender Dysphoria, Candidate for Hormones

Domains: Check all that apply

X Professionalism

X Communication and Interpersonal skills

X Medical History

- Physical exam
- Shared Decision Making

X Patient Education

X Clinical Reasoning

- Documentation
- Handoff
- Presentation
- Other:

Type and level of learner: Family Medicine Resident or Intern

Case Objectives: Competency Language

1. Gather essential and accurate information about patients and their conditions through history taking, physical examination, review of prior data and health records, laboratory data, imaging and other tests.

2. Partners/personalizes healthcare services, prevention and promotion of health

3. Communicates with patients/public with diverse socioeconomic/cultural backgrounds

4. Humanism/cultural proficiency with diverse patients

Education Objectives:

By the end of this activity, learners will be able to:

1. Use patient’s affirmed name and pronoun

2. Demonstrate a history and exam relevant to gender-affirming care

3. Formulate a hormone prescribing and monitoring plan with your preceptor

4. Execute an informed consent for hormones and shared decision making on a prescribing plan with your patient

| SETTING: | Family Medicine outpatient office |
| --- | --- |
| PATIENT PROFILE: | |
| Age range | 21-49 |
| Religious/spiritual background | All may be used |
| Sex (e.g., male, female, intersex, transwoman, transman, nonbinary) | All may be used (please see gender below) |
| Sexual Orientation (e.g., heterosexual, lesbian, gay, bisexual, pansexual, queer, asexual) | All may be used |
| Gender expression (e.g., man, woman, gender queer) | Nonbinary, they/them pronouns |
| Race/ethnicity: | All may be used |
| Physical description (e.g., BMI, height range) | All may be used |
| Physical limitations | All may be used |
| Patient appearance (e.g., disheveled, hospital gown, business casual, casual) | Work scrubs |
| Moulage + location (e.g., none, bruises, scars, body piercing, tattoos) | All may be used |
| Affect (e.g., pleasant, cooperative) | Nervous, worried |
| Family group (e.g., who is family, who they live with) | Lives with partner Sam, nonbinary who has had a hysterectomy. Co-parenting 2 children. |
| Education | Finished high school and training as dental hygienist |
| Level of health literacy | High |
| Employment, if any - present and past, noting any current stresses | Dental Hygienist |
| Home/homeless - type of dwelling, number of stories, owned or rented | Safe home with family group above, all may be used |
| Financial situation- any current stresses | Recently got health insurance with new job, no other stressors |
| Insurance Status (e.g., un/under/insured, public/private, HMO/PPO) | New insurance, all may be used. |
| Habits (i.e., diet, exercise, caffeine, smoking, alcohol, drugs) | No nicotine, 2 drinks of alcohol a month, some caffeine, no other substances. Irregular exercise and diet is ok. |
| Activities (i.e., hobbies, sports, clubs, friends) | All may be used |
| Typical day - what is the usual daily routine | All may be used |

| CASE INFORMATION | |
| --- | --- |
| Chief Concern: What the patient will say when greeted by the student. The patient’s primary reason for seeking medical care often stated in his/own words. | I’m here today because I finally got health insurance with my new job and want to start working with a provider on getting hormones. I heard from a friend this is a safe place to talk about it and where they have been prescribed hormones. |
| Additional Concerns: Other, if any, concerns the patient has today (i.e., symptoms, requests, expectations, etc.) that will become part of set agenda. | None |
|  | |
| THE PATIENT STORY: The SP will be asked to tell their symptom story and the personal and emotion impact for each of their concerns. You will want to write this is the patient voice. The symptom story should be able to answer this question: “Tell me more about [chief concern/additional concern], starting at the beginning and bringing me up to now.”  The personal context should be able to answer questions concerning the broader personal/psychosocial context of symptoms, especially the patient beliefs/attributions.  The emotional context should be able to ask how are you doing with this, how does this make you feel, how has this affected you emotionally? IMPACT: How has this affected your life? How has this been for your family? | I have felt something was unaligned with my designated sex since I was in elementary school, but only became familiar with the concept of gender transition about 10 years ago. I have been working with a therapist for the past few months and am confident in my gender identity and desire to start medical transition. I have discussed my plans with my partner Sam who is also non-binary, my family, and my friends who are supportive.  I identify as non-binary and use they/them pronouns.  I am not currently taking gender-affirming hormones and have not taken them in the past. I am interested in starting hormones as soon as possible. I have close friends who have transitioned with hormones and don’t have any specific questions, but heard other friends have had bad experiences with healthcare providers.  I would like to start hormones therapy at the lowest recommended dose. |
| HISTORY OF PRESENT ILLNESS: Although some of the HPI will be given in the patient’s symptom story, the learners will expand the story during the direct question section. Below describe the detailed history, usually about the chief concern, which the student must develop in order to make a useful assessment of the problem: | |
|  | |
| Onset (when; gradual or sudden) | n/a |
| Setting (what was going on or where was patient when symptoms first noticed?) | n/a |
| Duration (how long) | Since elementary school |
| Time relationships (frequency, constant or intermittent) | Constant |
| Location | n/a |
| Radiation | n/a |
| Quality | Severe |
| Amount | n/a |
| Aggravated by what | n/a |
| Relieved by what | n/a |
| Associated with what | n/a |
| Attitude (what does the patient think is the problem, and how does he/she feel about it) | They are confident in their “diagnosis” and plan. |
| Overall course | Identified in childhood and confident in need to start hormones. |
| REVIEW OF SYSTEMS: Significant positives and negatives | |
| Positive for anxiety, well controlled without medication | Negative for chest pain |
| Negative for depression, self harm, suicidal ideation | Negative for shortness of breath |
| No leg swelling | No rash |
|  |  |
|  | |
| Past medical history | Anxiety |
| Medication allergies (Name and reaction) | None |
| Environmental allergies (Name and reaction) | None |
| Illnesses | None |
| Vaccinations | Up to date |
| Surgeries | None |
| Accidents/ injuries/ trauma | None |
| Hospitalization | None |
|  | |
| Inclusive sexual and reproductive history | |
| Sexual practices  Sexual partners  Protection: Use of safer sex practices  Use of birth control if appropriate  Risk of intimate partner violence | Has sex with one nonbinary partner who has a vagina and has had a hysterectomy. Does not use protection and does not need birth control. No current or prior intimate partner violence. |
| Ob/GYN HISTORY (SP chooses to identify if assigned male or female at birth, so both histories provided)- | Designated female at birth: never been pregnant, not planning to get pregnant or have another child. Menses in teenage years without abnormality.  Designated male at birth: partner does not have a uterus so no plan for additional children. |
| Medications | Prescription/dose/reason – none  Over the counter/dose/reason - none  Herbs/supplements/dose/reason - multivitamin for wellness and energy |
| Immunizations | X Tetanus  X Flu   - Hepatitis - Pneumovax   X HPV   - Other |
| Tobacco products:   - Cigarettes - Cigar - Pipe - Chew - E-cigarettes | X Never   - Past- year started/year quit - Current   - Quantity   - # of years |
| Alcohol  X Beer  X Wine   - Liquor - Other | - Never - Past- year started/year quit   XCurrent   - - Quantity: 2 per month   - # of years: 10 years |
| Drugs   - Weed - Cocaine - Heroin - Meth - Other - IV - Inhalants - Other | X Never   - Past- year started/year quit - Current   - Quantity - # of years |
| Diet (describe) | Regular without limitations – any may be used |
| Exercise (describe) | Try to do cardio regularly for anxiety |
| List any other important social history or information important to this case | As above |
| Family history |  |
| Mother, Father, Siblings, Grandparents, and other significant findings. | NO history of heart disease, stroke, cancer, blood clots, high blood pressure, cholesterol or diabetes. |
|  |  |
| Physical Exam- List exam maneuvers expected for this case and any abnormal findings that SP will simulate. (tenderness, hyper-hypo reflex, rebound, weakness etc. ) | |
| PHYSICAL EXAM FINDINGS |  |
| 1. Written in layman’s terms | SP will give participant a card when asked for exam |
| 1. General appearance- affect, appearance, position of patient at opening (i.e. sitting, laying down, holding abdomen etc.) | No distress, pleasant, somewhat anxious appearing normal head and neck, lungs clear, heart regular without murmurs, stomach normal, extremities warm without swelling, no abnormal neurological findings |
| 1. Vital signs | Not yet available |
| 1. Specific findings and affect | Anxious appearing |
| 1. Response to certain physical movements | N/A |
|  |  |
| DIAGNOSIS AND DIFFERENTIAL |  |
| Diagnosis with support from positive and negative history and PE findings | Gender dysphoria, transgender, or gender non-binary |
| Differential with support from positive and negative history and PE findings | N/A |
|  |  |
| MANAGEMENT OR DIAGNOSTIC PLAN | Perform informed consent for first prescription of hormones, either testosterone or estrogen+spironolactone (per SP preference). No need for mental health assessment for prescription. Labs today for basic metabolic panel, serum estradiol and total testosterone, and additionally a blood count if selecting testosterone. Consider any required health maintenance labs, and if not done previously, STI screening. Follow up in 3 months. |
|  |  |
| PROFESSIONALISM ISSUES OR CHALLENGES: | Using wrong pronouns, not listening to patient’s concerns, open communication, saying yes to hormones without proper discussion of informed consent. |
