# Supplementary material for: An Effective Gender-Affirming Care and Hormone Prescribing Standardized Patient Case for Residents
Source: MedEdPORTAL. 2022 Jun 3;18:11258. doi: 10.15766/mep_2374-8265.11258 (PMC9163229; doi:10.15766/mep_2374-8265.11258)
Supplement: Supplementary file 1 — Standardized Patient Case Development Tool.docxStandardized Patient Case Scenario.docxParticipant Case Materials.docxObserver Checklist.docxPhysical Exam Results.docxPre-Post Survey.docx [file mep_2374-8265.11258-s001.zip › F. Pre-Post Survey.docx]

Transgender Health Standardized Patient Case Scenario Pre/Post Survey

| KNOWLEDGE / SKILL | **Extremely unconfident**  **(1)** | **Not very**  **confident**  **(2)** | **Unsure**  **(3)** | **Somewhat**  **Confident**  **(4)** | **Very**  **Confident**  **(5)** |
| --- | --- | --- | --- | --- | --- |
| **How confident are you to…** |  |  |  |  |  |
| Define the important terms relating to gender identity, sexual orientation, and gender dysphoria |  |  |  |  |  |
| Ask preferred name and pronouns |  |  |  |  |  |
| Effectively communicate with transgender individuals in a safe and respectful manner |  |  |  |  |  |
| Evaluate a patient for gender dysphoria |  |  |  |  |  |
| Evaluate whether it is safe and appropriate to start an individual on hormone based therapy |  |  |  |  |  |
| Perform an informed consent for initiating hormone based therapy. |  |  |  |  |  |
| Start an individual on hormone based therapy |  |  |  |  |  |

| **AWARENESS** | **Strongly**  **Disagree**  **(1)** | **Disagree**  **(2)** | **Neutral**  **(3)** | **Agree**  **(4)** | **Strongly**  **Agree**  **(5)** |
| --- | --- | --- | --- | --- | --- |
| **I feel that…** |  |  |  |  |  |
| I can describe health disparities that affect the transgender community |  |  |  |  |  |
| I can identify barriers transgender individuals would encounter in my clinic environment |  |  |  |  |  |
| I know the transition-related care and resources available in my health system |  |  |  |  |  |
| **ATTITUDE** | **Strongly**  **Disagree**  **(1)** | **Disagree**  **(2)** | **Neutral**  **(3)** | **Agree**  **(4)** | **Strongly**  **Agree**  **(5)** |
| **I think…** |  |  |  |  |  |
| Primary care practitioners should offer transition related care for transgender individuals |  |  |  |  |  |
| Offering transition related care in primary care will help decrease health care disparities for transgender individuals |  |  |  |  |  |
| Primary care practitioners have a responsibility to help transgender individuals navigate the health system to help address their health needs |  |  |  |  |  |
| Primary care practitioners have a role in creating a safe and welcoming environment for transgender individuals in the health system |  |  |  |  |  |

| **INTENDED**  **BEHAVIOR** | **Not at all likely (1)** | **Not very likely (2)** | **Impartial (3)** | **Likely (4)** | **Very Likely (5)** |
| --- | --- | --- | --- | --- | --- |
| **How likely are you to…** |  |  |  |  |  |
| Provide transition related care for your transgender patients? |  |  |  |  |  |
| Engage in activities to make my clinic space more welcoming to transgender individuals? |  |  |  |  |  |
| Seek out additional learning opportunities to improve my care of transgender individuals? |  |  |  |  |  |
